# Design methodology for low-power, low-voltage inductor-less Chua's chaotic oscillator

**DOI:** 10.1016/j.mex.2025.103365

**Published:** 2025-05-17

**Authors:** Chandan Kumar Choubey, Amit Gupta, Aruna Pathak

**Affiliations:** a Symbiosis Institute of Technology, Pune Campus, Symbiosis International (Deemed University), Pune, Maharashtra, India; b Department of Information Technology, Indian Institute of Information Technology, Bhopal, India; c Department of Electronics and Communication Engineering, Government Engineering College, Bharatpur, Rajasthan, India

**Keywords:** Chua’s circuit, Chaotic signal, Low power circuit, Non-linear circuit, DXCCII, DTMOS Technology, Design methodology for low-power, low-voltage, inductorless Chua's chaotic oscillator using DTMOS-based DXCCII.

## Abstract

This paper describes the design and modeling of a low-power, low-voltage Chua's chaotic oscillator using Dynamic Threshold Metal Oxide Semiconductor (DTMOS) technology. The primary active block used for this design is the DTMOS-based Dual-X Second-Generation Current Conveyor (DXCCII), which is an efficient current-mode active block that dramatically improves circuit performance and efficiency. The nonlinear active element of the Chua circuit, also called the Chua diode, is built using a single DXCCII block. Also, a synthetic inductor using only one DXCCII is used instead of a real inductor is used, which eases the fabrication process. As a result, the complete circuit requires only two DXCCIIs, allowing for an inductor-free design. The suggested circuits, including the Chua diode and Chua circuits, are simulated in PSPICE utilizing TSMC 180 nm CMOS technology. A DTMOS implementation of the DXCCII has been realized and utilized in the simulation with 180 nm CMOS technology. The slopes and the breakpoints of the nonlinear resistors are obtained from the V-I characteristics of Chua's diode, which confirms the theoretical and mathematical predictions. Attractors and chaotic waveforms obtained in the simulation validate the proposed DTMOS-based Chua chaotic oscillator. The circuit uses a bias voltage of ±0.2 V and consumes only 1.58 µW.

• A low-power, low-voltage design of Chua's chaotic oscillator using DTMOS technology has been proposed in this paper.
• With only two DTMOS-based DXCCIIs, an inductor-less design has been implemented.
• The proposed circuit uses a bias voltage of ±0.2 V and consumes only 1.58 µW.

## Graphical abstract

**Figure:**
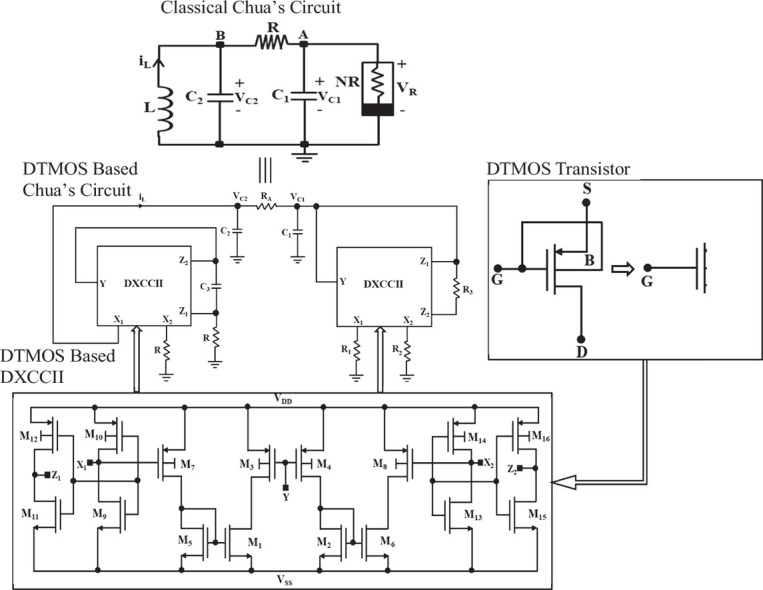

Specifications table

**Table:** 

Subject area:	Engineering
More specific subject area:	Electronics circuit design
Name of your method:	Design methodology for low-power, low-voltage, inductorless Chua's chaotic oscillator using DTMOS-based DXCCII.
Name and reference of original method	M. P. Kennedy, Robust op-amp realization of Chua's circuit, Frequenz, (46) (1992).https://doi.org/10.1515/FREQ.1992.46.3–4.66
Resource availability	NA

## Background

**Fig. 1:**
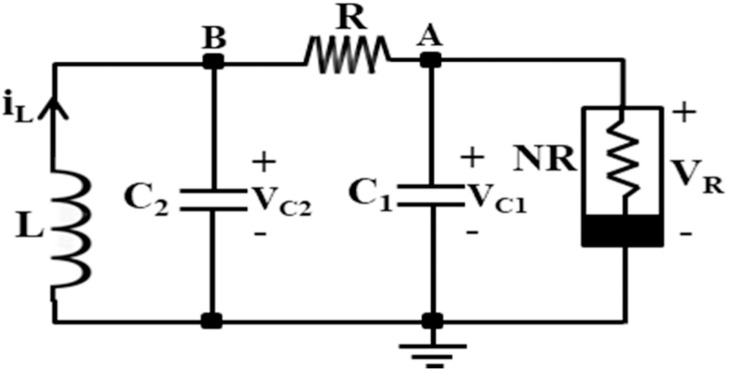
The traditional Chua's circuit [6].

The Chua circuit is the simplest electronic chaotic circuits that exhibit chaotic behavior. A chaotic circuit can be understood as a circuit whose outputs are highly sensitive to the initial conditions and seem to be random despite being generated by certain deterministic principles. Many natural phenomena, like weather, seismic activity, biological systems, and wind flow, exhibit chaotic behavior [[1], [2], [3], [4], [5]]. Therefore, researchers who investigate non-electrical chaotic systems, like these natural phenomena, first try to design an electrical chaotic system and do some experiments in the electrical lab [6]. Following this trend, in 1983, Leon. O. Chua investigated the simplest electric circuits with one non-linear resistor, two capacitors, one inductor, and one variable resistor, as shown in Fig. 1 [6].

Like another chaotic circuit, a system of differential equations controls the Chua circuit, given in (1), (2), and (3), that exhibit deterministic but unexpected behaviour [6].

(1) $$\frac{dV_{C1}}{dt}=\frac{1}{R_{A}C_{1}}\left(V_{C1}-V_{C2}\right)-\frac{1}{C_{1}}f\left(V_{R}\right)$$

(2) $$\frac{dV_{C2}}{dt}=\frac{1}{R_{A}C_{2}}\left(V_{C1}-V_{C2}\right)-\frac{1}{C_{2}}i_{L}$$

(3) $$\frac{di_{L}}{dt}=-\frac{1}{L}V_{C2}$$

Where i_L,_ V_C1_, and V_C2_ are current through inductor L and voltages across capacitors C_1_ and C_2_, respectively, as indicated in Fig. 1, and F(V_R_) is a nonlinear function defined as the current through the Chua's diode as [6]:

(4) $$f\left(V_{R}\right)=i_{R}=m_{0}V_{R}+0.5\left(m_{1}-m_{0}\right)\times \left[\left|V_{R}+V_{BP}\right|-\left|V_{R}-V_{BP}\right|\right]$$

**Fig. 2:**
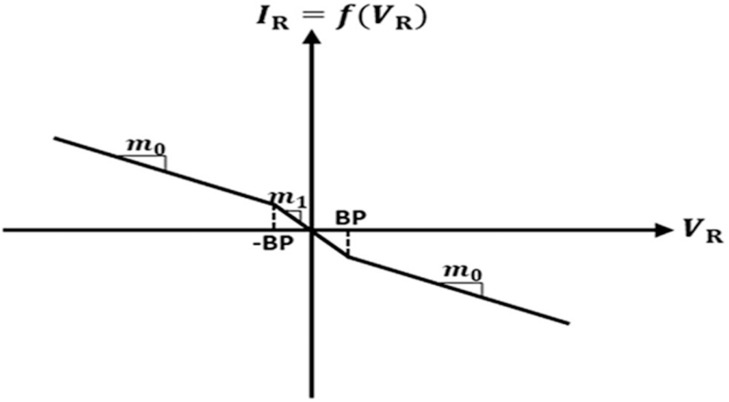
V-I Characteristic of NR [6].

Here, *m*_1_, *m*_0_, and *V_BP_* are the inner slope, outer slope, and breakpoint, respectively, as marked in Fig. 2.

*The traditional* Chua's circuit essentially uses a characteristic equation for a third-order oscillator, which *was obtained using the routine analysis as given in [**7**]:*

(5) $$s^{3}+s^{2}\left[\frac{1}{R_{A}C_{2}}+\frac{\frac{1}{R_{A}}+m_{i}}{C_{1}}\right]+s\left[\frac{1}{LC_{2}}+\frac{m_{i}}{R_{A}C_{1}C_{2}}\right]+\left[\frac{\frac{1}{R_{A}}+m_{i}}{LC_{1}C_{2}}\right]=0$$

From (5), the FO (frequency oscillation) and CO (condition of oscillation) of the Chua's circuit can be obtained as in [*7*]:

(6) $$FO:f_{0}=\frac{1}{2\pi \sqrt{LC_{2}}}\sqrt{1+\frac{Lm_{i}}{R_{A}C_{1}}}$$

(7) $$CO:R_{A}=-\frac{Lm_{i}\left(C_{1}+C_{2}\right)}{{C_{1}}^{2}+LC_{2}{m_{i}}^{2}}$$

A constraint $|\frac{Lm_{i}}{R_{A}C_{1}}|<1$ can be observed in (6). It gives two instantaneous oscillating frequencies for *i* = 0 and 1 under two different linear approximations. One of them becomes the dominant frequency on the spectrum.

Many authors attempted to design Chua's circuit using different ABBs (Analog Building Blocks) like Op-Amp (Operational Amplifier) [8,9], CCII (Second Generation Current Conveyor) [10], VDGA (Voltage-Differencing Gain Amplifier) [11], CCCTA (Current Controlled Current Conveyor Transconductance Amplifier) [12], CCCFA (Current-Controlled Current Conveyor Feedback Amplifier) [13], CDTA (Current Differential Transconductance Amplifier) [14], EXCCCII (Extra-X Current Controlled Current Conveyor) [15], DVCCTA (Current Conveyor Transconductance Amplifier)[16], OTRA (Operational Transresistance Amplifier) [17,18], Current Conveyor Transconductance Amplifier (CCTA) [19], VDTA (Voltage Differencing Transconductance Amplifier) [7], and several more. Some claim that the inductor-less design is because they use a synthetic inductor instead of a real inductor, as a real inductor is problematic in chip fabrication as it takes up more area [20,21]. In contrast, other uses real inductors. In both approaches, authors have approached in a standard way that the vital element of the Chua's circuit, i.e., the Chua's diode, has been designed using various types of ABBs and few resistors to make it simple.

The above-mentioned literature clarifies Chua's circuit as it is implementable with a variety of active building blocks. A current-mode building block that is highly versatile and useful is the dual-X second-generation current conveyor (DXCCII). DXCCII provides a blend of high performance, versatility, high linearity, ease of integration, and power efficiency, making it highly suitable for advanced analog signal processing tasks [[22], [23], [24], [25], [26], [27], [28], [29], [30], [31]]. Its ability to handle wide bandwidths, maintain low noise levels, and operate efficiently under various conditions underscores its importance in modern electronic design.

Low-voltage operation requires a lowering in the threshold voltage; however, conventional CMOS technologies in the future will not see a decrease in the threshold voltage with the supply voltage [32]. The dynamic threshold voltage MOSFET (DTMOS), which was first described in 1994 [33], is an obvious solution, and many low-supply voltage circuits have been presented [34].

In this work, a low-power, low-voltage novel inductor-less Chua circuit has been designed using DTMOS-based DXCCII for biomedical applications. Portable biomedical equipment's usability and efficacy depend heavily on its capacity to operate at minimal power. These devices' low power consumption enables them to function for prolonged periods of time without requiring frequent recharging. Also, numerous biomedical equipment are utilized in remote or home care environments with limited access to power sources. These gadgets can operate efficiently in a variety of settings, attributable to their low power consumption.

First, the critical element, Chua's diode, is created using a single DXCCII and three resistors. It is designed using the proper combination of two NRs (negative resistances). The combination of two NRs makes Chua's diode a nonlinear element. Then, a synthetic inductor is designed using one DXCCII, two resistors, and one capacitor. Finally, Chua's circuit has been developed using the DXCCII-based Chua's diode and synthetic inductor, two capacitors, and one resistor.

By filling up the study gaps in the literature, the proposed Fast Chaotic Oscillator's research invention symbolizes the circuit's uniqueness. The originality of the circuit is highlighted by a few important aspects. This work presents an innovative design of Chua's chaotic oscillator and Chua's diode utilizing just one DXCCII block.

The novel Chua's chaotic oscillator operates on low-voltage ±0.2 V. The proposed Chua's chaotic oscillator achieves a low power consumption of only 1.58µW. Its low power consumption makes it perfect for biomedical wearables and Internet of Things applications, as it prolongs the battery life of portable devices. Overall, this work enhances the understanding of chaotic circuits and opens up new avenues for integrating such systems into biomedical technologies, paving the way for more efficient diagnostic tools and innovative healthcare solutions. The formation of chaotic attractors with the simulation findings additionally confirms the circuit's competence, i.e., I-periodic attractor, double-scroll attractor, large limit cycle, and Rossler-type attractor, confirming mathematical and theoretical expectations.

## Method details

In this section, we start a discussion with the low-voltage, low-power DTMOS technology and the main active building block of the proposed circuit, DXCCII, followed by the implementation of Chua's diode based on DXCCII.

### DTMOS transistor

**Fig. 3:**
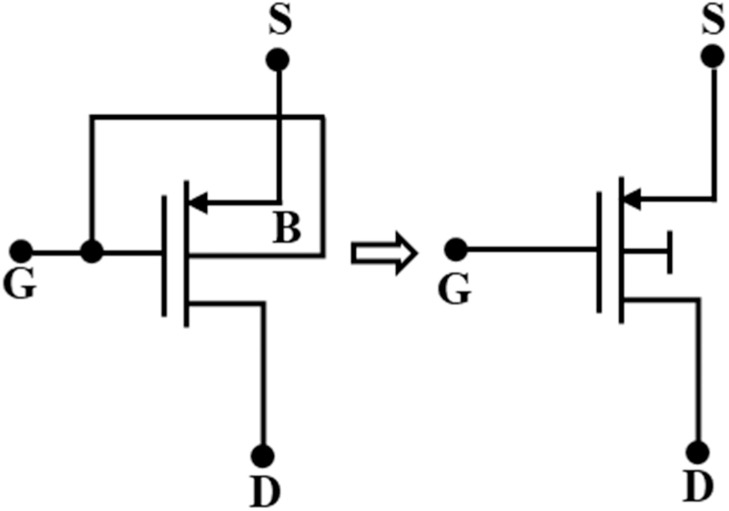
DTMOS transistor and symbol.

Assederaghi et al. originally proposed the DTMOS transistor for Silicon-On-Insulator (SOI) manufacturing technology in their pioneering research [33]. A device whose gate is connected to its substrate is a DTMOS transistor, as shown in Fig 3.

As a result, the device can be considered as a dynamically controlled threshold PMOS or as a linear bipolar p-n-p, as shown in (8); the substrate voltage in DTMOS varies in response to changes in the gate input voltage and causes a change in threshold voltage (V_TH_) accordingly.

(8) $$V_{TH}=\;V_{TO}+\gamma (\sqrt{\left|2\;\phi_{0}\right|+V_{SB\;}}-\;\sqrt{\left|2\;\phi_{0}\right|}$$

where γ is the body effect factor, and ϕ_0_ is the total surface band bending. The zero-bias threshold voltage is V_TO_, while the source-to-body voltage is represented by V_SB_. Standard digital technology only allows for the implementation of p-type DTMOS, as n-wells are controlled. The V_SB_ is either positive or zero for traditional MOS functioning, but it can also become negative for DTMOS operation. Nevertheless, the equation in (8) still holds true for somewhat negative values as long as the junction currents are negligibly tiny [35].

A PMOS-based DTMOS transistor exhibits the same subthreshold slope, off-current, and V_TH_ as a conventional PMOS if the voltage applied at the gate is high, causing the transistor to transition to the off state. The V_TH_ lowers if the input voltage drops, indicating that the transistor is in an ON state. Additionally, the bulk-to-source junction voltage is forward-biased at this time. The body effect causes a more significant source-to-drain current than in cases of typical PMOS. Because of this phenomenon, the DTMOS transistor is the preferred option for subthreshold operating regions because it doesn't require any more space and allows us to utilize the entire input range [36]. As power-saving designs that do not require high-frequency operation, the subthreshold mode of CMOS operation is typically chosen. Furthermore, because of their gate-to-body linked structure, DTMOS that operate in the subthreshold region have properties that are comparable to those of lateral bipolar p-n-p transistors but without the need for a comparatively high base current and with less flicker noise than typical MOS transistors [36].

A significant issue with DTMOS designs is the lateral bipolar transistor, which has drain body and source body junctions that have the potential to latch up and create extremely elevated body currents. It needs to be controlled to ensure that the device operates properly. In modern heavily doped substrates, the mobile carrier concentrations at supply voltages in the range of 0.4–0.5 V cannot attain high levels, resulting in source body and drain body junctions that have elevated turn-on voltages [34]. Therefore, if the supply voltages are near 0.4–0.5 V, forward-bias diode currents have no impact on the transistor's functioning or the design as a whole, as demonstrated experimentally in circuitry realizations [34].

Therefore, it can be claimed that DTMOS transistors are appropriate for low-voltage, low-power applications when operating in the subthreshold region.

### DTMOS-based DXCCII

**Fig. 4:**
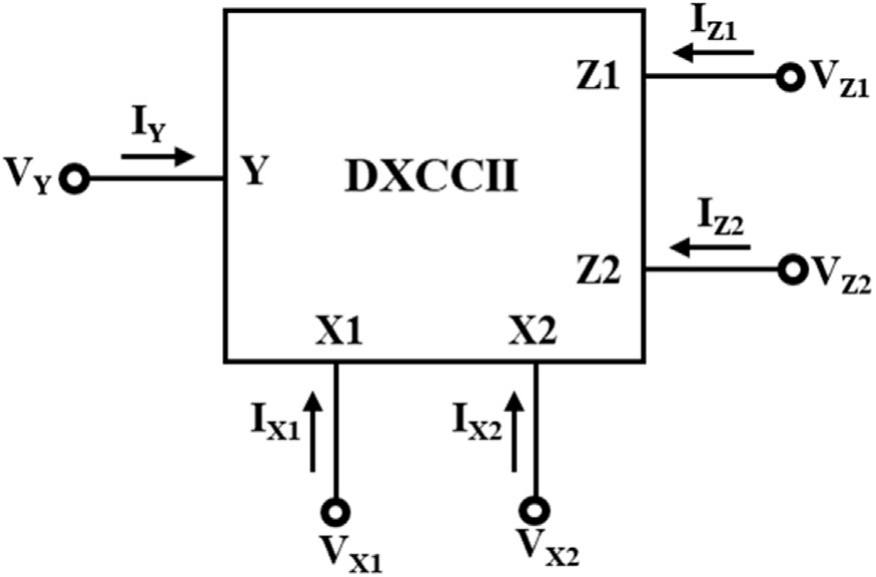
An electrical symbol of DXCCII.

**Fig. 5:**
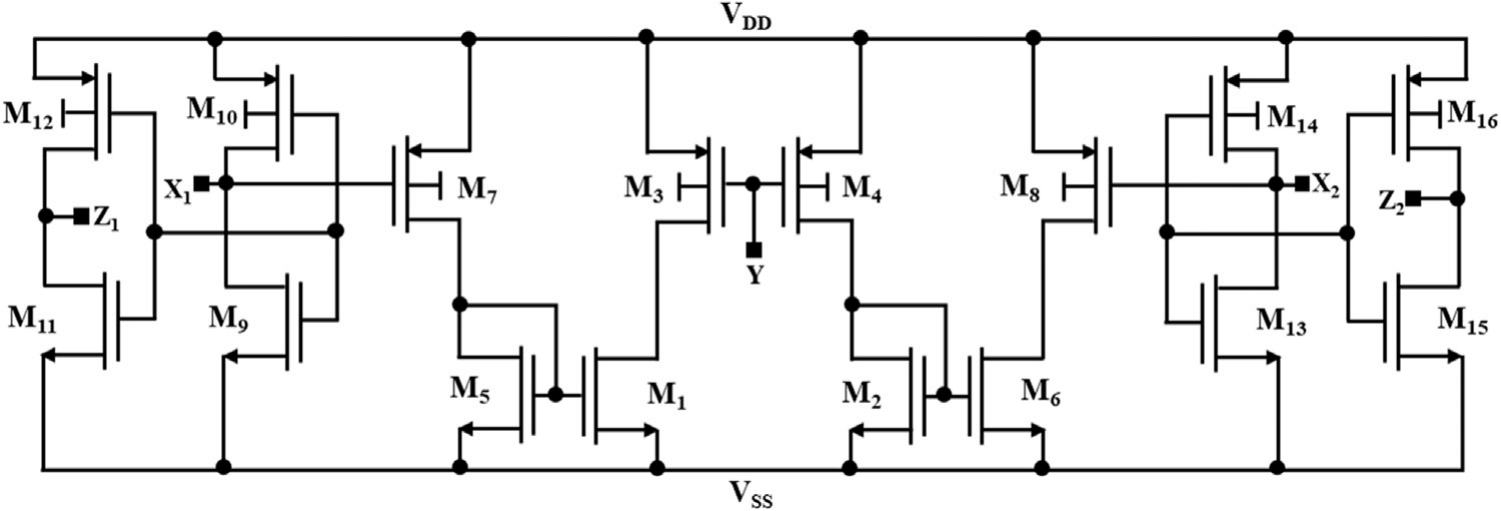
A CMOS implementation of DXCCII.

DXCCII is a combination of two CCIIs (second-generation current conveyors). The electrical symbol of DXCCII is shown in Fig. 4 [23]. It is a five-terminal ABB. There is one high-impedance input terminal, Y, two low-impedance terminals, X_1_ and X_2_, and two output terminals, Z_1_ and Z_2_. The terminal relationships are given in (9). From (9), one can observe that the input voltage V_Y_ appears at terminals X_1_ and X_2_ as V_X1_ and V_X2_, respectively [23]. Further, the current I_X1_ and I_X2_ are conveyed at the terminal Z_1_ and Z_2_ separately as I_Z1_ and I_Z2_, respectively. Also, the DTMOS-based schematic of DXCCII is shown in Fig. 5 [23].

(9) $$\left[\begin{array}{c} \begin{array}{c} I_{Y}\\ V_{X1}\\ V_{X2} \end{array}\\ I_{Z1}\\ I_{Z2} \end{array}\right]=\left[\begin{array}{c} 0\\ 1\\ -1\\ 0\\ 0 \end{array}\;\begin{array}{c} 0\\ 0\\ 0\\ 1\\ 0 \end{array}\;\begin{array}{c} 0\\ 0\\ 0\\ 0\\ 1 \end{array}\right]\;\left[\begin{array}{c} V_{Y}\\ I_{X1}\\ I_{X2} \end{array}\right]$$

The transistors in the circuit structure function in the subthreshold region. The supply voltages in Fig. 4 proposed DTMOS-based DXCCII circuit are set at ± 0.2 V. There is a maximum bias voltage of 0.4 V across the transistors. latch-up issues brought on by excessive diode currents are avoided. Table 1 lists the transistor aspect ratios for the circuit shown in Fig. 5. MOS circuits that operate in the subthreshold region at such low supply voltages and significant transistor size allow the targeted current to flow under extremely low voltage conditions; hence, in the design, transistors that are relatively large are employed.

**Table 1:** NMOS and PMOS transistor aspect ratios utilized in Fig. 5.

Transistors	PMOS Transistors								NMOS Transistors							
	M3	M4	M7	M8	M10	M12	M14	M16	M1	M2	M5	M6	M9	M11	M13	M15
Width (W) in µm	300	300	300	300	300	300	300	300	50	50	50	50	320	320	320	320
Length (L) in µm	2	2	2	2	2	2	2	2	2	2	2	2	0.4	0.4	0.4	0.4

### Chua's diode employing a single DTMOS-based DXCCII

The proposed Chua's diode is designed by amalgamating negative resistances, NR_1_ and NR_2_. The design and the mathematical expressions for NR_1_ and NR_2_ are as follows.

### Design of NR_1_

**Fig. 6:**
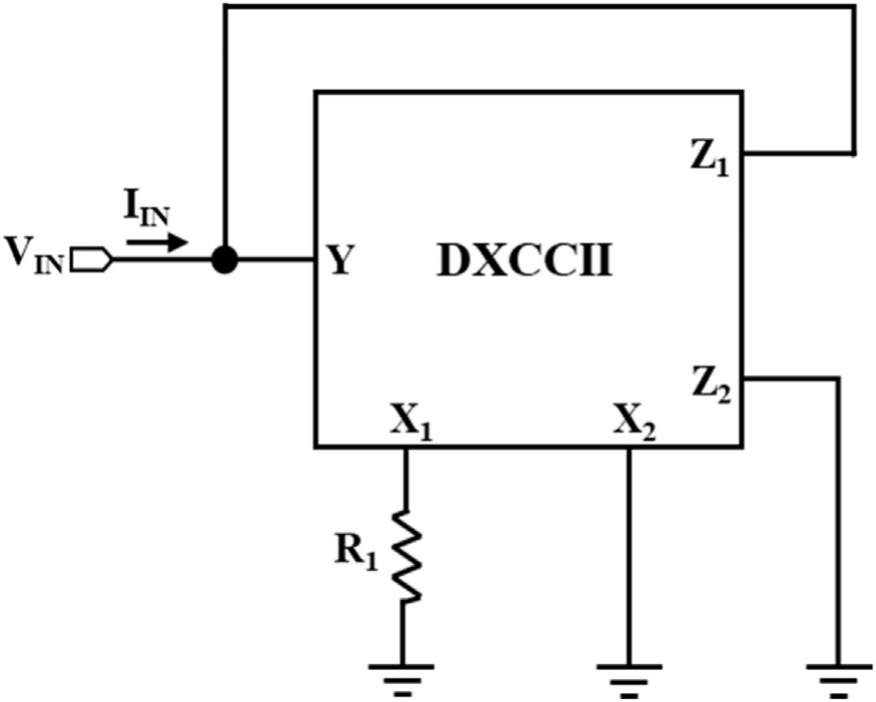
NR_1_ design using a single DTMOS-based DXCCII.

The design of NR_1_ using a single DTMOS-based DXCCII and a single resistor is illustrated in Fig. 6. By using the port-relationship of the DXCCII, given in (9), the internal slope (m_11_), the outer slope (m_01_), and the breakpoints are described as:

(10) $$m_{11}=\;\frac{V_{IN}}{I_{IN}}\;=\;-\frac{1}{R_{1}}$$

(11) $$m_{01}\;\approx \;\infty$$

(12) $$\pm V_{BP1}\;\approx \;\pm V_{sat}$$

### Design of NR_2_

**Fig. 7:**
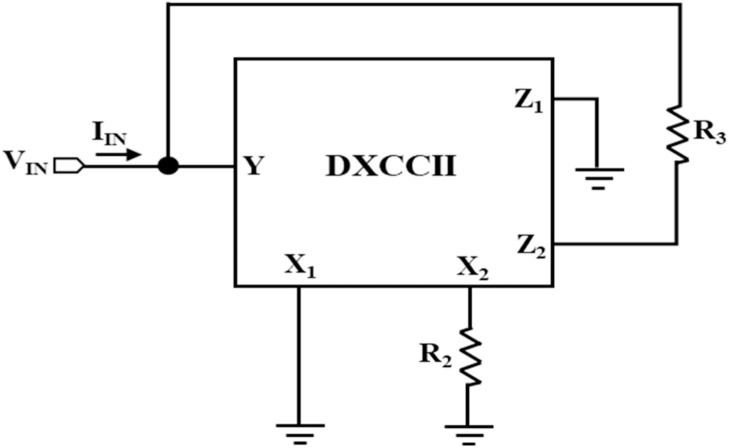
NR_2_ design using a single DTMOS-based DXCCII.

The design of NR_2_ using a single DTMOS-based DXCCII and a single resistor is illustrated in Fig. 7. By using the port-relationship of the DXCCII, given in (9), the internal slope (m_11_), the outer slope (m_01_), and the breakpoints are described as:

(13) $$m_{12}=\;\frac{V_{IN}}{I_{IN}}\;=\;-\frac{1}{R_{2}}$$

(14) $$m_{02}=\;\frac{1}{R_{3}}$$

(15) $$\pm V_{BP2}=\frac{\pm V_{sat}}{1+\;\frac{R_{3}}{R_{2}}}$$

### Design of Chua's diode

**Fig. 8:**
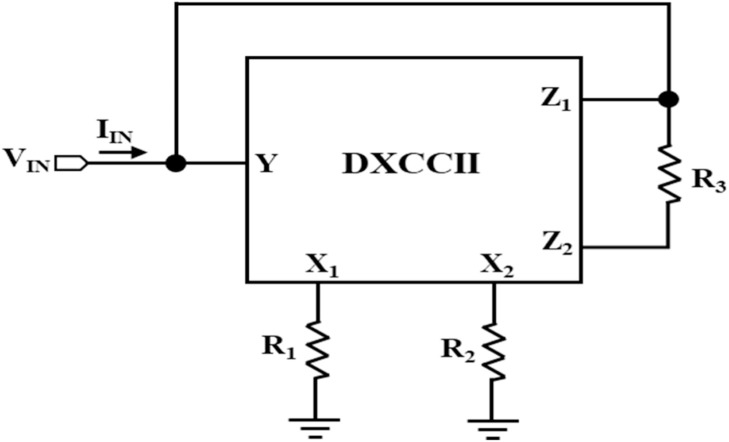
Chua diode implementation using a single DXCCII.

Now, by amalgamating negative resistances, NR_1_ and NR_2_, a compact Chua's diode is designed using a single DTMOS-based DXCCII, as shown in Fig. 8. It consists of three resistors. The following parameters of the Chua's diode can be seen in its V-I characteristics, illustrated in Fig. 2: (i) inner slope (m_1_), (ii) outer slope (m_0_), and breakpoint voltages (V_BP1_ and V_BP2_). The expressions for these parameters are obtained by using the routine analysis as follows:

(16) $$m_{1}=\;\frac{V_{IN}}{I_{IN}}=\;-\frac{1}{R_{1}}-\;\frac{1}{R_{2}}=\;m_{11}+\;m_{12}$$

(17) $$m_{0}=\;\frac{V_{IN}}{I_{IN}}=\;-\frac{1}{R_{1}}+\;\frac{1}{R_{3}}=\;m_{11}+\;m_{02}$$

(18) $$\pm V_{BP1}=\;\pm V_{sat}\text{and}\pm V_{BP2}=\;\frac{\pm V_{sat}}{1+\;\frac{R_{3}}{R_{2}}}$$

Where m_11_ and m_12_ are the inner slopes of the NR_1_ and NR_2_, respectively, m_02_ is the outer slope of the NR_2_, and ±V_sat_ is the saturation point voltages.

### Synthetic inductor using a single DTMOS-based DXCCII

**Fig. 9:**
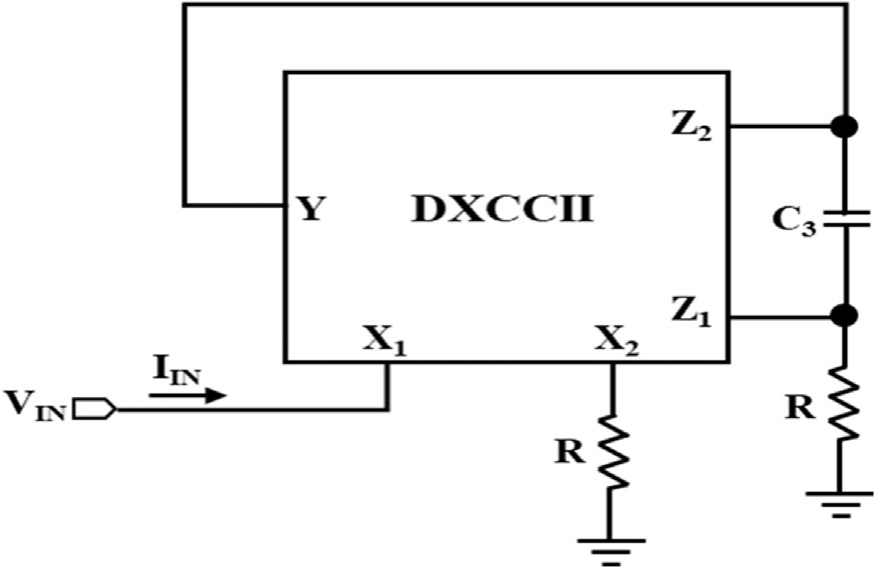
Synthetic inductor using a single DTMOS-based DXCCII.

A DXCCII-based synthetic inductor is utilized to make Chua's circuit inductor-less. The synthetic inductor circuit, shown in Fig. 9, uses one DTMOS-based DXCCII, one capacitor, and two resistors. The expressions for the input impedance (Z_IN_) and the equivalent inductance (L_eq_) are obtained by the routine analysis of the synthetic inductor circuit as given in (19) and (20), respectively.

(19) $$Z_{IN}=\;\frac{V_{IN}}{I_{IN}}=sC_{3}R^{2}$$

(20) $$L_{eq}=\;C_{3}R^{2}$$

### Inductor-less Chua's circuit

**Fig. 10:**
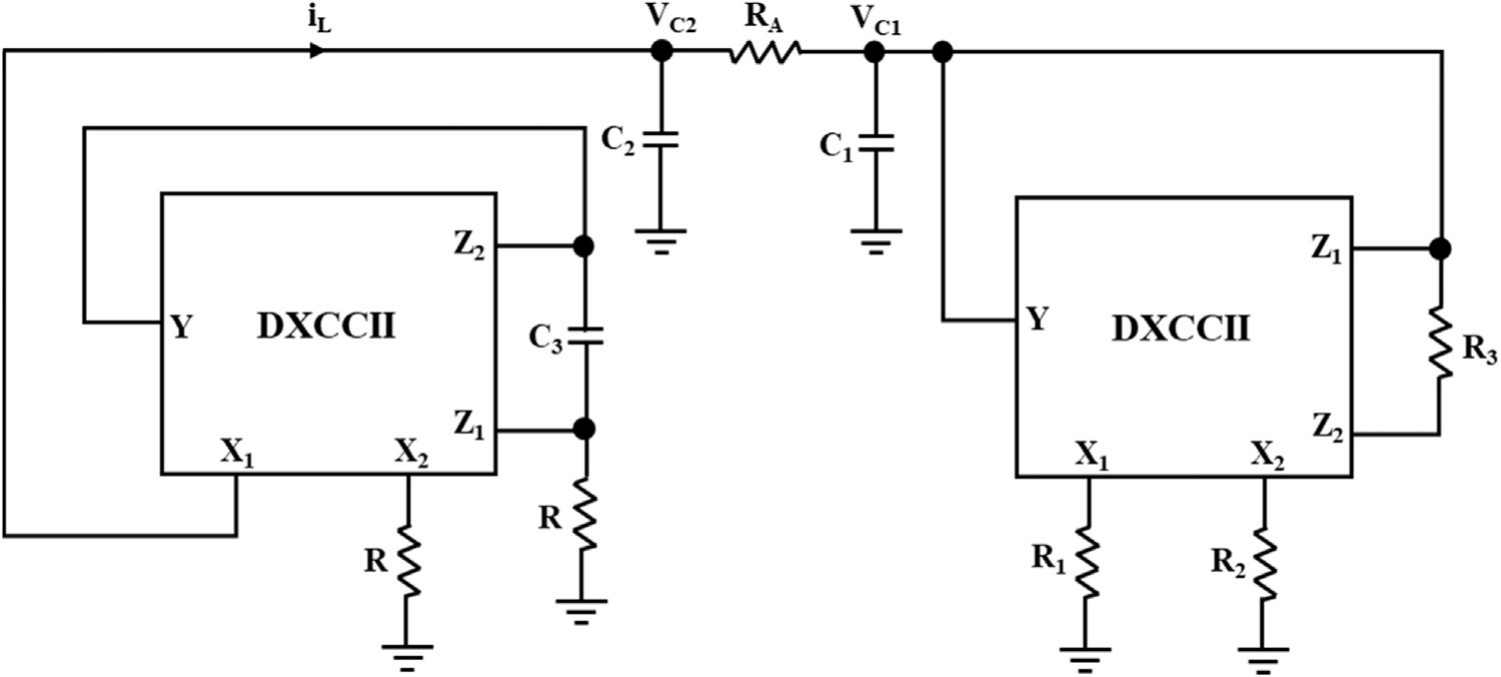
Proposed inductor-less low-power Chua's Circuit.

The conventional Chua's circuit, illustrated in Fig. 1, is now implemented using a DXCCII-based Chua's diode in Fig. 8 and a synthetic inductor in Fig. 9. The proposed Chua's circuit is illustrated in Fig. 10. As stated in the former section, Chua's circuit is a third-order chaotic oscillator. It can generate various chaotic signals while operating in sustained oscillation mode with a fixed set of values for Chua's diode parameters. Also, the set of values for inductor L, capacitors C_1_ and C_2_, and resistance R_A_ determines the circuit's operating frequency. This circuit's chaotic signals can be obtained as voltages across capacitors C_1_ and C_2_, i.e., V_C1_ and V_C2_, and current through inductor L, i_L_.

### Method validation

The proposed low-voltage, low-power Chua chaotic circuit is simulated in PSPICE to check the operating performance of this circuit. DTMOS-based schematic of DXCCII, shown in Fig. 5, is drawn and simulated in 0.18 µm CMOS technology node.

To implement this, a ±0.2V power supply was utilized, along with the aspect ratios (W/L) provided in Table 1. It is found that the circuit consumes only 1.58µW. In biomedical applications, low-power operation is crucial to ensuring that devices are useful, dependable, and easy to use, which will ultimately improve healthcare outcomes.

**Fig. 11:**
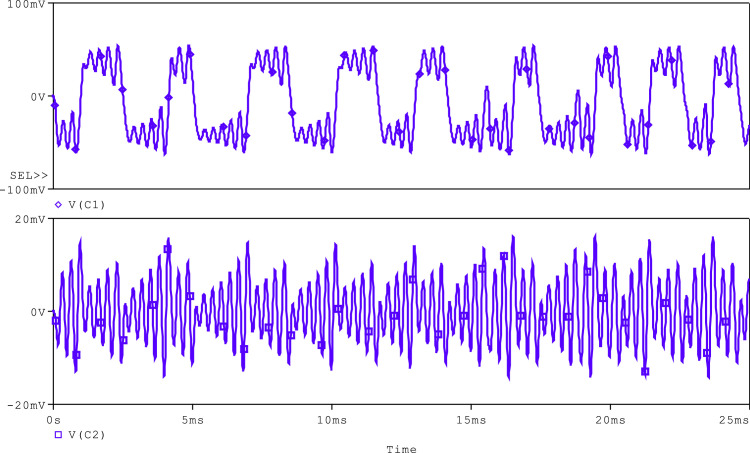
Voltage chaotic waveforms across the capacitors C_1_ and C_2_ of Fig. 10.

**Fig. 12:**
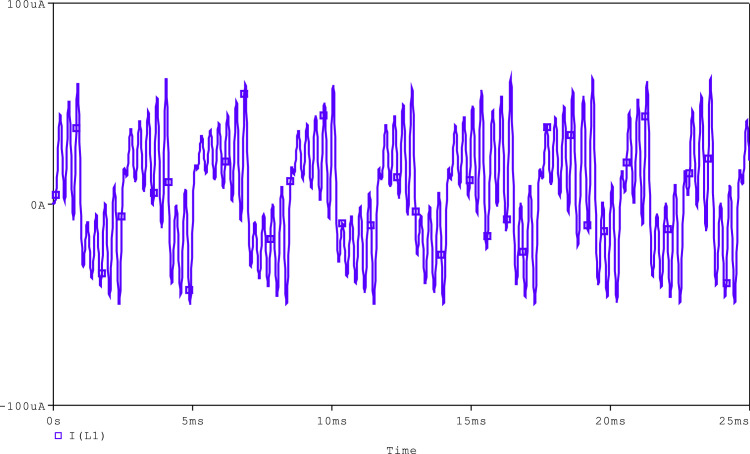
Current chaotic waveforms through the inductor L of Fig. 10.

In the simulation of Chua's circuit, shown in Fig. 10, the synthetic inductor circuit is tuned for the equivalent inductance of 90mH by using *R* = 1kΩ and C_3_ = 19nF. Chua's diode, which was discussed above, has been used. The values of capacitors, C_1_ and C_2_, are taken as 10 pF and 100 pF, respectively. Resistors, R_1_, R_2_, and R_3_, of this circuit are taken as 1.8kΩ, 2.9kΩ, and 22kΩ, respectively. As illustrated in Figs. 11 and 12, the generated chaotic signals are obtained as V_C1_, V_C2_, and i_L_, respectively.

**Fig. 13:**
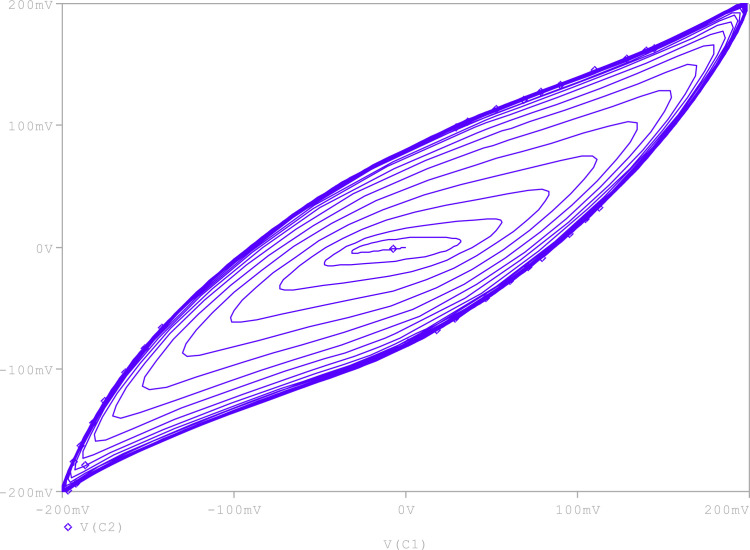
Large limit cycle obtained at R_A_ = 1300Ω.

**Fig. 14:**
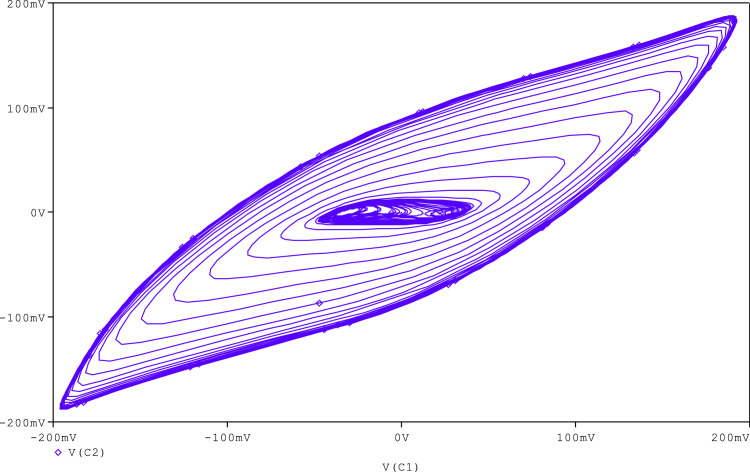
Large limit cycle obtained at R_A_ = 1500 Ω.

**Fig. 15:**
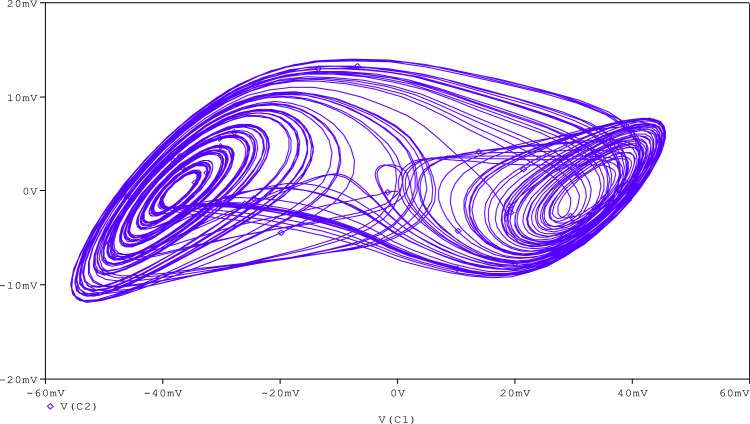
Double scroll attractor obtained at R_A_ = 1600 Ω.

**Fig. 16:**
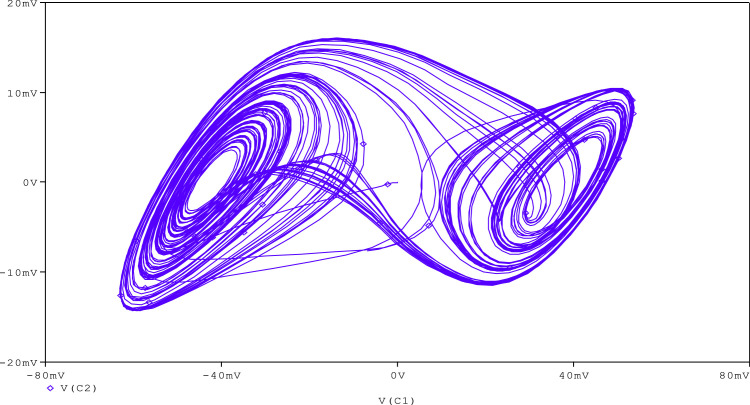
Double scroll attractor obtained at R_A_ = 1700 Ω.

**Fig. 17:**
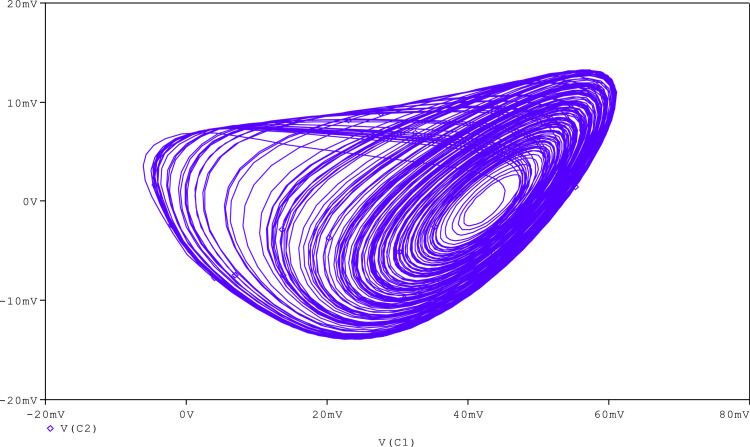
Rossler type attractor obtained at R_A_ = 1800 Ω.

**Fig. 18:**
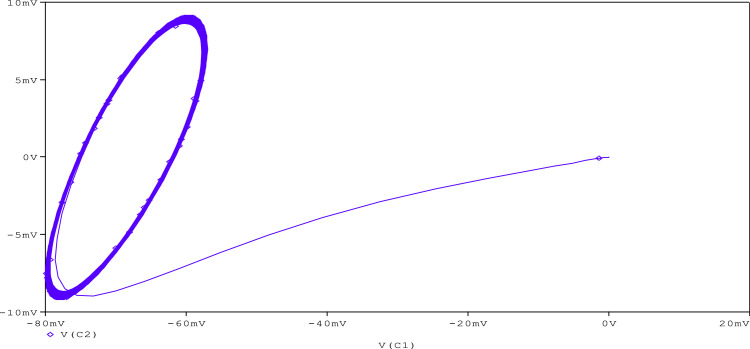
I-periodic attractor obtained at R_A_ = 2230 Ω.

Further, to check the robustness of the proposed circuit, the resistance (R_A_) is tuned to get various chaotic attractors. Furthermore, by altering the R_A_ for 1300Ω, 1500Ω, 1600Ω, 1700Ω,1800Ω, and 2230Ω, respectively, chaotic attractor types such as large limit cycle, double-scroll attractor, Rossler-type attractor, and I-periodic attractor can be produced as shown in Fig. 13, Fig. 14, Fig. 15, Fig. 16, Fig. 17, Fig. 18, which further indicates the proposed chaotic oscillator circuit's capability.

### Comparison

This section compares the proposed work with the various implementations of Chua's circuit that are available in the literature. A precise comparison is presented in Table 2. Comparison is made on the basis of the number of active building blocks used, the technology node used, the number of passive elements used, inductors implementation, the power supply voltage, and the power consumption. It is observed that the proposed circuit uses the minimum power supply voltage (± 0.2 V) in comparison to the available implementations of Chua's chaotic oscillators. Over and above, the proposed circuit reports the lowest power consumption (1.58µW) among available implementations of Chua's chaotic oscillators.

**Table 2:** Comparison table of various implementations of Chua's circuit.

Ref. No.	Authors, Year	No. of Active Building Blocks used	Technology node used	No. of Passive Elements used (R + C + L)	Inductorless implementation	Power Supply Voltage	Power Consumption
6	Kennedy MP, 1992	2, OA	BJT based IC	7R+2C+1L	NO	±9V	Not Given
10	Zhijun et al., 2017	11, CCII	BJT based IC	19R+3C	NO	+9V	245mW
11	Choubey & Paul, 2022	2,VDGA	0.18 µm CMOS	3C	YES	±0.9 V	Not Given
15	Bhatt, et al., 2022	1, EXCCCII	CMOS 0.25 μm	1 R + 3C	YES	±1.25 V	Not Given
16	Kushwaha & Paul, 2016	3, DVCCTA	CMOS 0.25 µm	6R+3C	YES	±1.5V	Not Given
17	Kushwaha & Paul, 2016	3, OTRA	CMOS 0.5 µm	10R+4C	YES	±1.5V	Not Given
31	Choubey & Paul, 2020	1, VDTA	CMOS 0.18 µm	2R+2C+1L	NO	±0.9V	0.243mW
–	Proposed work	2, DXCCII	CMOS 0.18 µm	6R+3C	YES	±0.2V	1.58µW

## Conclusion

A low-power and low-voltage Chua chaotic oscillator is implemented with DXCCII, employing DTMOS transistors to generate chaotic waveforms. Initially, a simple Chua diode known as a non-linear resistor is constructed using a single DXCCII and three resistors. Further, the synthetic inductor is implemented with one DXCCII, one capacitor, and two resistors. This Chua diode and synthetic inductor are further used in the Chua chaotic oscillator. A CMOS implementation with 180 nm technology simulates the proposed Chua diode and chaotic oscillator. The simulation yields three chaotic waveforms: V_C1_, V_C2_, and I_L_. A variety of chaotic attractors are additionally produced with the chaotic waveforms, indicating that the proposed circuit can produce sustained chaotic waveforms. The proposed chaotic oscillator makes a substantial contribution and reduces the complexity of design, with less implementation of the inductor and with minimum components. Furthermore, it exhibits low power consumption (1.58µW) and is capable of operating under low (± 0.2 V) symmetric supply voltages. The proposed circuit has several potential real-world applications demanding portable chaos systems, including biomedical applications.

## Limitations

Not applicable.

## Ethics statements

This research did not involve research on human subjects or animal experiments, and no data is involved from social media platforms.

## CRediT author statement

**Chandan Kumar Choubey**: Conceptualization, Software, Validation, Funding acquisition, Writing - original draft. **Amit Gupta:**Writing - review & editing, Validation. **Aruna Pathak**: Writing - review & editing, Supervision.

## Declaration of Competing Interest

The authors declare that they have no known competing financial interests or personal relationships that could have appeared to influence the work reported in this paper.

## Data Availability

No data was used for the research described in the article.
